# The prevalence of hypovitaminosis D and its risk factors in pregnant women and their newborns in the Middle East: A systematic review

**DOI:** 10.18502/ijrm.v17i10.5284

**Published:** 2019-11-07

**Authors:** Shayesteh Hajizadeh, Judy Rankin Shary, Susan Gayle Reed, Carol Lynn Wagner

**Affiliations:** Department of Pediatrics, Medical University of South Carolina, Charleston, SC.

**Keywords:** Vitamin D, Pregnancy, Newborns, Cord blood, Middle East.

## Abstract

**Background:**

Pregnant women and newborns are at risk for vitamin D deficiency (VDD). Also, poor health outcomes for pregnant women with VDD are reported in the published literature.

**Objective:**

The aim of this systematic review was to estimate the prevalence of hypovitaminosis D and the associated risk factors for hypovitaminosis D in Middle Eastern pregnant women and their newborns.

**Results:**

The prevalence of circulating 25-hydroxyvitamin D (25(OH)D) < 50 nmol/L as a marker of vitamin D status in pregnant women and their newborns was between 24.5-98% and 22-100%, respectively. The prevalence of 25(OH) D < 25 nmol/L in pregnant women and their newborns was over a wide range between 16.7-80% and 22-82%, respectively. Predictors for low maternal and neonatal 25(OH)D concentrations included decreased vitamin D synthesis due to reduced exposure to sunlight and decreased nutritional intake of vitamin D. A predictor of low neonatal 25(OH)D concentrations included maternal vitamin D status and the correlation between vitamin D concentrations in maternal and cord blood.

**Conclusion:**

The high prevalence of VDD in the pregnant women of the Middle East underscores the necessity of implementing national prevention and intervention strategies. A clear policy for clinicians and healthcare workers is needed for screening and maintaining sufficient vitamin D status during pregnancy.

## 1. Introduction 

Vitamin D deficiency (VDD) is a global public health problem in all age groups (1). There are four recent meta-analyses showing that pregnant women and newborns are at a risk for VDD (2-5). A number of studies have reported that pregnancy alone increases the risk of VDD (6). Results of two recent meta-analyses showed that there was a significant relationship between maternal VDD and adverse maternal, fetal, and postnatal outcomes (7, 8).

Poor health outcomes for pregnant women with VDD have been reported and include: gestational diabetes mellitus (7-11), preeclampsia (7-9, 12-14), intrahepatic cholestasis of pregnancy (15), preterm labor (16, 17), cesarean section delivery (18-20), periodontal disease (21, 22), and human immunodeficiency virus (HIV) progression (23). Poor health outcomes for newborns as a consequence of maternal VDD have also been reported, including increased risk of low birth weight mainly due to prematurity (7), small-for-gestational-age (7-9, 20, 24-27), body composition and cardiovascular disease risk factors in the offspring (28, 29), abnormal skeletal development (30-35), abnormal immune development (24, 26, 27, 30, 36-38), affected respiratory health including wheezing and asthma (24, 27, 30, 36, 39, 40), type 1 diabetes (26, 27, 30, 34-36), and abnormal neurocognitive development in childhood (41). VDD rickets occurs most commonly during early infancy and is prevalent in infants of mothers who have poor vitamin D stores (42).

The countries of the Middle East - Bahrain, Cyprus, Egypt, Iran, Iraq, Israel, Jordan, Kuwait, Lebanon, Oman, Palestine, Qatar, Saudi Arabia, Syria, Turkey, United Arab Emirates, and Yemen - have a high incidence of VDD and rickets

(43-44). Although the Middle East has a hot, sunny and arid climate; and is located within the latitudes from 12N-42 N; both of which allow for vitamin D synthesis from ultraviolet B (UVB) rays for most months of the year and for more than 8 hr/day (45), there exists significant VDD. Therefore it is important to determine the mother's vitamin D status and associated factors during pregnancy in order to prevent neonatal VDD and related complications (46-48).

A number of recent reviews have included the rapidly growing body of literature on vitamin D during pregnancy. However, because few studies conducted in the Middle East and North Africa were population-based, extrapolation to the vitamin D nutritional status of pregnant women and their neonates in that region is limited (44). In the past five yr, only one review focused on maternal and newborn vitamin D status, and in this review, just 13% of 95 studies were from countries of the Eastern Mediterranean which includes the countries of the Middle East and Afghanistan, Djibouti, Libya, Morocco, Pakistan, Somalia, Sudan, Tunisia, and not Bahrain, Israel, Occupied Palestinian territories, and Greece (49).

To address this lack of a review representing the Middle East specifically, we undertook a systematic review of the literature on hypovitaminosis D and risk factors in pregnant women and their newborns in the countries of the Middle East.

## 2. Materials and Methods 

This systematic review was conducted following the Preferred Reporting Items for Systematic Reviews and Meta-Analyses reporting guidelines.

### Search strategy

This study is a systematic review of the published literature about vitamin D status in pregnant women and their newborns in the Middle East. It was conducted by exploring international electronic databases PubMed and Scopus with the following MeSH and Entry Terms: (“Infants, Newborn” OR “Newborn Infant” OR “Newborn Infants” OR “Newborns” OR “Newborn” OR “Neonate” OR “Neonates” OR “Women, Pregnant” OR “Pregnant Woman” OR “Woman, Pregnant” OR “Blood, Fetal” OR “Bloods, Fetal” OR “Fetal Bloods” OR “Cord Blood” OR “Blood, Cord” OR “Bloods, Cord” OR “Cord Bloods “OR” Umbilical Cord Blood” OR “Blood, Umbilical Cord” OR “Bloods, Umbilical Cord” OR “Cord Blood, Umbilical” OR “Cord Bloods, Umbilical” OR “Umbilical Cord Bloods”) AND (“Vitamin D” OR “Deficiency, Vitamin D” OR “Deficiencies, Vitamin D” OR “Vitamin D Deficiencies”) AND (“Middle East” OR “Near East” OR “Arab Countries” OR “Palestine” OR “Bahrain” OR “Iran” OR “Iraq” OR “Israel” OR “Jordan” OR “Kuwait” OR “Lebanon” OR “Egypt” OR “Cyprus” OR “Oman” OR “Qatar” OR “Saudi Arabia” OR “Syria” OR “Turkey” OR “United Arab Emirates” OR “Yemen”).

The period of publication was from 2000 to 2017. Publications were searched and reference lists were hand-searched. The confirmed sources were examined using a data extraction form. Based on the protocol, we reviewed all cross-sectional studies, prospective studies, and case-control studies, which had been conducted on the status of vitamin D in pregnant women and their newborns in the Middle East in the desired time limit.

### The process of study selection 

**Figure 1:**
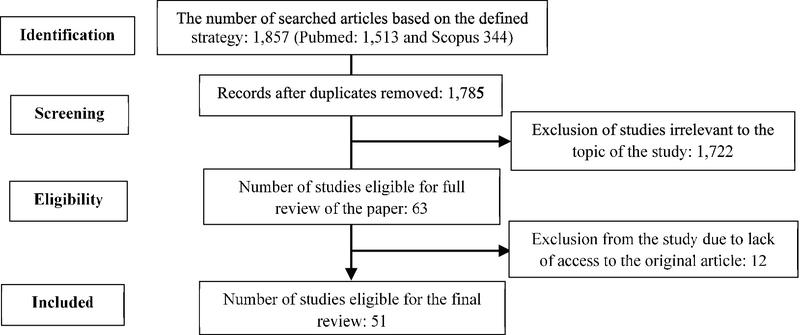
Identification of studies.

Study selection was performed in three phases: In the first phase, titles were scanned according to the selection criteria. The accepted titles were entered into the abstract review phase to identify studies for eligibility. In the next phase, abstracts were reviewed; the study was excluded if the reviewer found that the study met one or more exclusion criterion/criteria. The last phase was performed to determine if the full texts should be included for data extraction. In total, 1,857 articles were investigated, of which, 1,722 papers were excluded due to the lack of consistency with the goals of the study; as for example, studies on the general population (teenage, children, men, or breastfeeding women), and studies that provided no new empirical data (reviews, editorial letters, and brief items). Moreover, 12 papers were ruled out due to lack of access to the original article; for instance, because of the type of language (Turkish or Arabic language). Finally, 51 papers were included in this study (Figure 1).

### Data extraction, data elements, and quality assessment 

Each study was evaluated using a data extraction form. We developed a data extraction form to collect key indicators of each study, including study design, definition of VDD and vitamin D insufficiency, as well as assays used and characteristics of the study population. We extracted data on these indicators as reported in the articles. We collected data from the included studies and organized the results in a table format. The study outcomes are presented in the Results section.

We assessed the study quality by using data reported on representativeness and validity. A study was considered representative if: (1) this feature of the study was explicitly addressed in the corresponding full-text article, or (2) any statement made by the authors suggested that the actual sample reflected the target population. Validity was evaluated using information about the (25(OH)D) measure (e.g., participation of the laboratory in the International Vitamin D Quality Assessment Scheme, DEQAS) (50).

At any point, any disagreement between reviewers was resolved by means of meeting and discussion among all authors to establish a consensus.

## 3. Results

Table I shows the studies conducted to estimate VDD for pregnant women and newborns in Iran, Turkey, and Arabic countries. Estimates are reported using various assay methodology for the measurement of circulating 25-hydroxyvitamin D (25(OH)D) concentration, which serves as the indicator of vitamin D status. This metabolite (25(OH)D) is difficult to measure, with large variations between methods and among laboratories even when the same methods are used (51). This review revealed that the prevalence of (25(OH)D) concentration < 50 nmol/L in pregnant women and

their newborns was between 24.5-98% and 22-100%, respectively. The prevalence of (25(OH)D) concentration < 25 nmol/L in pregnant women and their newborns was over a wide range between 16.7-80% and 22-82%, respectively (Table II).

A number of factors can limit the cutaneous production of vitamin D and dietary sources of vitamin D (52), especially in pregnant women (53). As shown in Tables II and III, predictors for low maternal and neonatal (25(OH)D) concentrations included decreased vitamin D synthesis, likely due to reduced exposure to sunlight and decreased nutritional intake of vitamin D.

**Table 1:** Methods for laboratory testing of Vitamin D status (25(OH)D concentration) in reviewed studies


**Assay**	**Studies**	**Total**
Enzyme-binding immunoassay	Aly *et al*., 2013 (47); Al-Shaikh *et al*., 2016 (54); Al-Faris, 2016 (55); Alfaleh *et al*., 2014 (56); Aljebory, 2013 (57); Al Kalbani *et al*., 2011 (58); Khalessi *et al*., 2015 (59); Ainy *et al*., 2006 (60); Asemi *et al*., 2010 (61); Zahediasl and Eyni., 2004 (62); Pehlivan *et al*., 2002 (63)	11
Radioligand-binding assay	Bassir *et al*., 2001 (64)	1
Radioimmunoassay	Salek *et al*., 2008 (65); Maghbooli *et al*., 2008 (66); Maghbooli *et al*., 2007 (67); Narchi *et al*., 2010 (68); GÜR *et al*., 2014b (69); Bener *et al*., 2013 (70); Molla *et al*., 2005 (71); Güven *et al*., 2012 (72); (Kelishadi *et al*., 2013) (73)	9
Enzyme-linked immunosorbent assay	Soheilykhah *et al*., 2010 (11); Kazemi *et al*., 2009 (74); Asadi *et al*., 2015 (75) Rostami *et al*., 2015 (76); Rahbar *et al*., 2015 (77); Akhlaghi *et al*., 2015 (78); Abbasian *et al*., 2016 (79); Khosravi and Entekhabi, 2016 (80); Mirbolouk *et al*., 2016 (81); Gur *et al*., 2014 (82); Al-Ajlan *et al*., 2015 (83); Aydogmus *et al*., 2015 (84); Gür *et al*., 2014a (85); Halicioglu *et al*., 2012 (86)	13
Electrochemiluminescence	Alp *et al*., 2016 (6); Hatami *et al*., 2014 (87); Jafarzadeh *et al*., 2015 (88); Shor *et al*., 2015 (89); Al Emadi and Hammoudeh, 2013 (90); Zuhur *et al*., 2013 (91); Yildiz *et al*., 2012 (92); Parlak *et al*., 2015 (93)	8
High-performance liquid chromatography	Güven *et al*., 2012 (72); Gunduz *et al*., 2016 (94); Ustuner *et al*., 2011 (95); Kılıcaslan *et al*., 2017 (96); Yılmaz *et al*., 2017 (97); Khuri-Bulos *et al*., 2014 (98); Narchi *et al*., 2011 (99); Ergür *et al*., 2009 (100)	8
Liquid chromatography-tandem mass spectrometry	Ates *et al*., 2016 (101)	1
	51

**Table 2:** Prevalence of hypovitaminosis D in pregnant women and their newborns in the Middle East


**Author, year, ref no**	**Year**	**Country-city**	**Latitude N**	**Seasons**	**Trimester of pregnancy**	**N**	**Maternal age, Years (X ± SD)**	**25(OH)D ng/mL or (nmol/L)**			**Type of study and predictors**
				**Mean ± SD**	**Vitamin D deficiency (VDD)**	**Vitamin D insufficiency (VID)**	
**Author, year, ref no**	**Year**	**Country-city**	**Latitude N**	**Seasons**	**Trimester of pregnancy**	**N**	**Maternal age, Years (X ± SD)**	**25(OH)D ng/mL or (nmol/L)**			**Type of study and predictors**
				**Mean ± SD**	**Vitamin D deficiency (VDD)**	**Vitamin D insufficiency (VID)**	
(Al-Shaikh *et al*., 2016) (54)	2014	Saudi Arabia-Riyadh	24.7	Winter & Spring	Third	1000	29.03 ± 5.7	Mothers: 30.5 ± 19.6 nmol/L	< 20 ng/mL (50 nmol/L) mothers: 86.4 %	Cross-sectional study
(Al-Faris, 2016) (55)	2010	Saudi Arabia-Riyadh	24.7	Spring	First	160	- Median: mothers: 49.9 nmol/L	< 20 ng/mL (50 nmol/L) mothers: 50%	Cross-sectional study: age group, educational level, sun exposure frequency, and daytime and daily practice of exercise were significantly associated with vitamin D status
(Shor *et al*., 2015) (89)	2011	Israel-Jerusalem	31.7	Spring	Third	208	28.3 ± 5.8	Mothers: 15.0 ± 51.7 ng/mL	< 20 ng/mL (50 nmol/L) mothers: 89.9% newborns: 88.5%	20-30 ng/mL (50-75nmol/L) mothers: 5.8% newborns: 5.8%	Cross-sectional study: a high correlation was found between maternal and fetal vitamin D serum concentrations (r= 0.85, p< 0.001)
(Al-Ajlan *et al*., 2015) (83)	- Saudi Arabia-Riyadh	24.7	- First	515	28.7 ± 6.1	Mothers: 19.1 ± 15.1 nmol/L	< 10 ng/mL (25 nmol/L) mothers: 68%	10-20 ng/mL (25-50 nmol/L) mothers: 26.2%	Cross-sectional study
(AlFaleh *et al*., 2014) (56)	2013	Saudi Arabia-Riyadh	24.7	Winter	Third	200	- -	< 10ng/mL (25 nmol/L) newborns: 59.5%	< 20 ng/mL (50 nmol/L) newborns: 28%	Cross-sectional study
(Khuri-Bulos *et al*., 2014) (98)	2010-2011	Jordan-Amman	31	All	Third	3,731	Median: 27	Newborns: 8.6 nmol/L	< 20 ng/mL (50 nmol/L) newborns: 94.1%	Prospective study: lower gestational age, maternal smoke exposure, birth during winter months were associated with lower infant vitamin D levels
(ALjebory, 2013) (57)	2012	Iraq-Baghdad	33.3	Spring & Summer & Autumn	Third	50	- Mothers: 40.6 nmol/L newborns: 44.9 nmol/L	< 20 ng/mL (50 nmol/L) mothers: 40% newborns: 22%	20-30 ng/mL (50-75 nmol/L) mothers: 38% newborns: 66%	Cross-sectional study: positive correlation between 25(OH)D concentration in maternal and cord blood (r= 0.762, p= 0.0001). Sunlight exposure
(Bener *et al*., 2013) (70)	2011	Qatar-Doha	25.28	All	Third	1,873		< 20 ng/mL (50 nmol/L) mothers: 48.4%	Prospective study
(Aly *et al*., 2013) (47)	2011	Saudi Arabia-Al Khafji	28.42	- Third	92	33 ± 6.2	Mothers: 46.6 ± 16.5 nmol/L newborns: 40.1 ± 18 nmol/L	< 12ng/mL (30 nmol/L) mothers:14.2%	12-20 ng/mL (30-50 nmol/L) mothers: 50%	Cross-sectional study: maternal serum 25(OH)D${}_{3}$ strongly correlated with cord blood 25(OH)D${}_{3}$ (r= 0.89, p= 0.01). Decreasing trends across the categories of 25(OH)D${}_{3}$ were found for lower social class, those living in rural areas with a history of inadequate sun exposure, and ultiparous women
(Al Emadi and Hammoudeh, 2013) (90)	2007-2010	Qatar-Doha	25.28	All	First	97	Range (22-37)	Mothers: 17.2 ng/mL	- -	Prospective study
(Narchi *et al*., 2011) (99)	2007	United Arab Emirates-Al-`Ain	24	Autumn	Third	27	- Mothers: 35.5 nmol/L newborns: 44.7 nmol/L	< 10ng/mL (25 nmol/L) mothers: 30% newborns: 22%	10-20 ng/mL (25-50 nmol/L) mothers: 48% newborns: 22%	Prospective study
(Al Kalbani *et al*., 2011) (58)	2010	Oman-Muscat	23.58	Spring & Summer	First & second	103		< 10 ng/mL (25 nmol/L) mothers: 34%	25-30 ng/mL (50-75 nmol/L) mothers: 65%	Prospective study
(Narchi *et al*., 2010) (68)	2007	United Arab Emirates-Al-Ain	24	Autumn	All	75	Median 27	Mothers: antenatal visit: 17.3 ± 10.5 ng/mL after delivery: 14.4 ± 9.8 ng/mL	< 10ng/mL (25 nmol/L) mothers: antenatal visit: 26% after delivery: 32%	10-20 ng/mL (25-50 nmol/L) Mothers: Antenatal visit: 42% After delivery: 45%	Prospective study
(Molla *et al*., 2005) (71)	2005	Kuwait-Kuwait city	29	- Third	214	27.5 ± 4.2	Mothers: 14.6 ± 10.7 ng/mL newborns: 8.2 ± 6.7 ng/mL	< 10ng/mL (25 nmol/L) mothers: 40% newborns: 60%	Cross-sectional study: the vitamin D status of the mothers and neonates were highly correlated (r= 0.790, p< 0.001)
(Kilicaslan et a., 2017) (96)	2014	Turkey-Konya	37.9	Winter	Third	100	26.75 ± 5.30	Mothers: 11.4 ± 6.2 ng/mL newborns: 8 ± 5.0 ng/mL	< 10ng/mL (25 nmol/L) mothers: 53% newborns: 70%	< 10-30 ng/mL (25-75 nmol/L) mothers: 47% newborns: 30%	Cross-sectional study: 25(OH)D concentrations were found to be higher in the women who had received vitamin D. In the cord blood, 86.4% of VDD was attributed to the VDD in the mother (R${}^{2}$= 0.864)
(Yilmaz *et al*., 2017) (97)	2014-2015	Turkey-Samsun	41.3	All	Third	750	- Newborns: 11.4 ± 10.2 ng/mL	< 20 ng/mL (50 nmol/L) newborns: 87%	< 20-30 ng/mL (50-75 nmol/L) newborns: 9%	Cross-sectional study
(Ates *et al*., 2016) (101)	2012-2014	Turkey-Istanbul	41	All	First	229	29.49 ± 4.879	Mothers: 13 ± 9.4 ng/mL	< 19ng/mL (47.5 nmol/L) mothers: 82.1%,	20-29 ng/mL (50-72.5 nmol/L) mothers:13.5%	Prospective study: a high prevalence of VDD was related to dress code, use of multivitamins and season at sampling
(Alp *et al*., 2016) (6)	2012-2013	Turkey-Erzurum	39.4	All	Third	81	29.9 ± 3.4	Mothers: 7.1 ± 6.5 ng/mL newborns 7.0 ± 6.6 ng/mL	5-15 ng/mL (12.5-37.5 nmol/L) mothers: 34.5% newborns: 30.9%	- Prospective study: strong positive correlation between maternal serum and umbilical cord 25(OH)D${}_{3}$ concentrations (r= 0.624; p< 0.001). Dressing style, lack of sunlight in the house
(Gunduz *et al*., 2016) (94)	2013	Turkey-Ankara	40	All	Third	92	30.4 ± 4.6	Mothers: 22.9 ± 16.2 ng/mL	< 20ng/mL (50 nmol/L) mothers: 55.4%	20-32 ng/mL (50-80 nmol/L) mothers: 22.6%	Cross-sectional study
(Parlak *et al*., 2015) (93)	2012-2013	Turkey-LSES cities	37.35	All	Third	97	27.1 ± 4.5	mothers: 5.0 ± 3.3 ng/mL newborns: 4.3 ± 2.4 ng/mL	< 20ng/mL (50 nmol/L) mothers: 98% newborns: 100%	Prospective study/ strong positive correlation between maternal serum and umbilical cord 25(OH)D${}_{3}$ levels (r= 0.735, p< 0.05). Covered dressing style, not receiving any vitamin D supplementation, and primigravida women
(Aydogmus *et al*., 2015) (84)	2013-2014	Turkey-Izmir	38.4	All	Third	152	- -	< 20 ng/mL (50 nmol/L) mothers: 44.6%	- Prospective study
(Gür *et al*., 2014a) (85)	2012	Turkey-İzmir and Erzurum-	38.4, 39.9	Summer & Autumn	Second	İzmir: 387 erzurum: 245	Izmir: 28.4 ± 4.5 erzurum: 29.1 ± 5.1	- ≤ 20 ng/mL (50 nmol/L) mothers: izmir: 34.5% erzurum: 75.5%	- Cross-sectional study: clothing style, fish consumption, seaside holiday duration, and 1,200 IU/day vitamin D replacement had an effect on 25(OH)D${}_{3}$ concentrations in pregnant subjects in İzmir, whereas only holiday duration and 1,200 IU/day vitamin D replacement affected 25(OH)D${}_{3}$ concentrations in Erzurum
(Gür *et al*., 2014) (82)	2012	Turkey-Izmir	38.4	Summer & Autumn	Second	208	28.5	Mothers: 22.4 ± 11.2 ng/mL	< 20 ng/mL (50 nmol/L) mothers: 51.5%	Prospective study
(GÜR *et al*., 2014b) (69)	2009-2010	Turkey-Ankara	39.9	Autumn & Winter	Second	99	- -	≤ 20 ng/mL (50 nmol/L) mothers: 62.6% newborns: 58.6%	21-29 ng/mL (52.5-72.5 nmol/L) mothers: 18.2% newborns: 15.2%	Cross-sectional study
(Zuhur *et al*., 2013) (91)	2010-2011	Turkey-Istanbul	41	All	Second	402	30.85 ± 5.6	Mothers: 33 ± 16.2 nmol/L	< 10 ng/mL (25 nmol/L) mothers: 35.6%	10-19.9 ng/mL (25-49.9 nmol/L) mothers: 48.8%	Cross-sectional study
(Güven *et al*., 2012) (72)	2008	Turkey-Ankara	40	Winter	Third	101	- -	Severe < 12 ng/mL (30 nmol/L) newborns: 32%	- Cross-sectional study
(Yildiz *et al*., 2012) (92)	- Turkey-Izmir	38.4	Winter	Third	250	- Mothers: 11.5 ± 5.9 ng/mL newborns: 10.9 ± 5.9 ng/mL	- -	Cross-sectional study: 25(OH)D concentrations of mothers and umbilical cord blood samples were found to be correlated (r= 0.548, p< 0.01)
(Ustuner *et al*., 2011a) (95)	2008-2009	Turkey-Ankara	40	Aurumn & Winter	Third	79	26.4 ± 5.5	Mothers: 12.0 ± 7.2 ng/mL	Severe < 10 ng/mL (25 nmol/L) mothers: 45.6%	10-32 ng/mL (25-80 nmol/L) mothers: 51.9%	Cross-sectional study: in patients who used multivitamin supplements, 25(OH)D concentrations were significantly higher (p < 0.046)
(Halicioglu *et al*., 2012) (86)	2008	Turkey-Izmir	38.4	Spring	Third	258	27.2 ± 4.9	Mothers: 11.5 ± 5.4 ng/mL newborns: 11.5 ± 6.8 ng/mL	≤ 10 ng/mL (25 nmol/L) mothers: 50.4% ≤ 20 ng/mL (50 nmol/L) mothers: 90.3%	Cross-sectional study: uncovered dressing style, sufficient consumption of dairy products, and multivitamin use during gestation
(Ergür *et al*., 2009) (100)	2003-2005	Turkey-Ankara	40	All	Third	70	29.7 ± 4.3	< 25ng/mL (62.5 nmol/L) mothers: 81.4% newborns: 97.2%	Case-control study
(Pehlivan *et al*., 2002) (63)	2000	Turkey-Kocaeli	40.8	Spring	Third	78	26.1 ± 5.1	Mothers: 17.5 ± 10.3 nmol/L	< 10ng/mL (25 nmol/L) mothers: 79.5%	10-16 ng/mL (25-40 nmol/L) mothers:15.3%	Cross-sectional study: the risk factors associated with low maternal 25(OH)D concentrations were low educational level, insufficient intake of vitamin D from the diet, and “covered” dressing habits
(Abbasian *et al*., 2016) (79)	2012-2013	Iran-Shahrood	36.4	Winter & Spring	Third	284	26.6 ± 5.3	< 8 ng/mL (20 nmol/L) mothers: 1.1% newborns: 2.5%	8-12 ng/mL (20-30 nmol/L) mothers: 60.2% newborns: 48.9%	Cross-sectional study: weak correlation between maternal serum and cord blood 25(OH)D concentrations (r= 0.12, p=0.053)
(Mirbolouk *et al*., 2016) (81)	2013-2014	Iran-Rasht	37.3	All	All	176	27.5 ± 4.6	Mothers:15.6 ± 9.8 ng/mL	< 20 ng/mL (50 nmol/L) mothers: 69%	20-30 ng/mL (50-75 nmol/L) mothers: 21%	Cross-sectional study: there was a positive correlation between 25(OH)D concentrations and vitamin D consumption as a supplementary one before pregnancy (r = 2.473, p= 0.001)
(Khosravi and Entekhabi, 2016) (80)	2015	Iran-Tehran	35.7	- Third	49	Mothers: 26.1 ± 8.4 ng/mL newborns: 17.2 ± 10.4 ng/mL	< 20 ng/mL (50 nmol/L) mothers: 24.5% newborns: 71.4%	20-30 ng/mL (50-75 nmol/L) mothers: 46.9% newborns: 12.2%	Cross-sectional study: serum 25OHD concentration of the mothers and their neonates were significantly correlated (r = 0.446, p < 0.001)
(Khosravi and Entekhabi, 2016) (80)	2015	Iran-Tehran	35.7	- Third	49	Mothers: 26.1 ± 8.4 ng/mL newborns: 17.2 ± 10.4 ng/mL	< 20 ng/mL (50 nmol/L) mothers: 24.5% newborns: 71.4%	20-30 ng/mL (50-75 nmol/L) mothers: 46.9% newborns: 12.2%	Cross-sectional study: serum 25OHD concentration of the mothers and their neonates were significantly correlated (r = 0.446, p < 0.001)
(Rostami *et al*., 2015) (76)	2014	Iran-Masjed Soleimam	32	Summer	First	1,581	28.8 ± 5.5	Mothers: 13.1 ± 6.4 ng/mL	< 20 ng/mL (50 nmol/L) mothers: 84.4%	Cross-sectional study: mean serum 25(OH)D concentrations was significantly associated with the duration of sun exposure, use of sunscreens, type of hijab, and type of dwelling (p < 0.0001)
(Akhlaghi *et al*., 2015) (78)	2013-2014	Iran-Mashhad	36.26	All	Third	190	27.6 ± 4.3	Mothers: 27.3 ± 4.0 ng/mL newborns: 14.9 ± 8 ng/mL	< 12 ng/mL(30 nmol/L) mothers: 33.2%	12-20 ng/mL (30-50 nmol/L) mothers: 52.1%	Cross-sectional study: insufficient sunlight exposure and maternal skin type were its main risk factor. Birth during winter months was associated with lower infant vitamin D levels
(Rahbar *et al*., 2015) (77)	2014	Iran-Semnan	35.2	- First	180	Mothers: 25.9 ± 18.0 ng/mL	≤ 10 ng/mL (25 nmol/L) mothers: 16.7%	11-32 ng/mL (27.5-80 nmol/L) mothers: 54.4%	Cross-sectional study
(Asadi *et al*., 2015) (75)	2012-2014	Iran-Tehran	35.7	All	Third	186	28.5 6 ± 6.0	Median mothers: 11.7 ± 0.12 ng/mL	< 20 ng/mL (50 nmol/L) mothers: 74.4%	20-30 ng/mL (50-75 nmol/L) mothers: 23.3%	Cross-sectional study
(Khalessi *et al*., 2015) (59)	2011-2012	Iran-Tehran	35.7	All	Third	102	26.2 ± 10.0	Mothers: 31.5 nmol/L	< 10 ng/mL (25 nmol/L) mothers: 48%	10-20 ng/mL (25-50 nmol/L) mothers: 27.5%	Cross-sectional study
(Jafarzadeh *et al*., 2015) (88)	- Iran-Shahr-e Kord	32.3	- First & second	First: 155 second: 64	27.4 ± 5.3	Mothers: first: 25.9 ± 45.6 ng/mL second: 24.1 ± 39.5 ng/mL	- -	Cross-sectional study
(Hatami *et al*., 2014) (87)	2012	Iran-Bushire	28.9	Spring & Summer	Third	100	27.6 ± 6.1	Mothers: 13.5 ± 10.78 ng/mL	< 20 ng/mL (50 nmol/L) mothers: 76%	20-30 ng/mL (50-75 nmol/L) mothers: 14%	Cross-sectional study
(Kelishadi *et al*., 2013) (73)	2013	Iran-Isfahan	32.65	- Third	100	- Median mother: 15.1 ng/mL newborns: 15.7ng/mL	- -	Cross-sectional study: the air quality had an inverse and independent association with 25(OH)D concentrations of mothers and their neonates.
(Soheilykhah *et al.*., 2010) (11)	2007-2009	Iran-Yazd	31.9	All	Second	204	27.4 ± 5.1	< 20 ng/mL (50 nmol/L) mothers: 78.4%	20-30 ng/mL (50-75 nmol/L) mothers: 10.3%	Case-control study
(Asemi *et al*., 2010) (61)	2008-2009	Iran-Kashan	32.98	All	Second & Third	147	- -	≤ 10 ng/mL (25 nmol/L) mothers: 35.2%	11-32 ng/mL (27.5-80 nmol/L) mothers: 60.6%	Cross-sectional study
(Kazemi *et al*., 2009) (74)	2005	Iran-Zanjan	36.6	Winter & Summer	Third	67	28.5 ± 5	Mothers: 19.4 ± 3.9 nmol/L newborns: 16.7 ± 2.9 nmol/L	< 10 ng/mL (25 nmol/L) mothers: 71% newborns: 67%	Cross-sectional study: A positive correlation was found between maternal and cord blood 25(OH)D concentration (r < 0.55, p < 0.001).
(Salek *et al*., 2008) (65)	2005	Iran-Isfahan	32.65	Summer	Third	88	25.5 ± 5.3	Mothers: 52.2 ± 35.6 ng/mL newborns: 27.4 ± 11.4 ng/mL	< 35 ng/mL (87.5 nmol/L) mothers: 26.1% < 26ng/mL (65 nmol/L) newborns: 53.4%	- Cross-sectional study
(Maghbooli *et al*., 2008) (66)	2005	Iran-Tehran	35.7	- Second	741	Mothers: 9.2 ± 7.3 ng/mL	< 10 ng/mL (25 nmol/L) mothers: 70.6%	10-13.9 ng/mL (25-34.9 nmol/L) Mothers: 15.9%	Cross-sectional study
(Maghbooli *et al*., 2007) (67)	2002	Iran-Tehran	35.7	Winter	Third	552	- Mothers: 27.8 ± 21.71 nmol/L newborns: 18.1 ± 11.6 nmol/L	< 14 ng/mL (35 nmol/L) mothers: 66.8% newborns: 93.3%	Cross-sectional study: A significant correlation between maternal and cord blood serum 25(OH)D concentrations.
(Ainy *et al*., 2006) (60)	2002-2003	Tehran-Iran	35.7	All	All	48	26.2 ± 5.0	- < 20 ng/mL (50 nmol/L) mothers: first trimester: 60% second trimester: 48% Third trimester: 47%	Prospective study
(Zahediasl and Eyni, 2004) (62)	2002	Iran-Tehran	35.7	- First	52	25.9 ± 5.0	- < 10 ng/mL (25 nmol/L) mothers: 59.6%	- Cross-sectional study
(Bassir *et al*., 2001) (64)	2001	Iran-Tehran	35.7	- Third	50	- Mothers: 12.8 ± 26 nmol/L newborns: 4.94 ± 9.4 nmol/L	< 10 ng/mL (25 nmol/L) mothers: 80% newborns: 82%	- Prospective study: A significant and positive correlation between maternal and cord serum 25-OHD concentrations (r= 0.88; p < 0.0001).

**Table 3:** Risk factors associated with low maternal and neonatal 25(OH)D concentrations in the Middle East


Maternal	Decreased vitamin D synthesis due to reduced exposure to sunlight	Lifestyle that decreases the time spent outdoors and use of sunscreens
	Personal factors	Cultural practices when clothing covers more of the body surface
			Air pollution
	Environmental factors	Season
		Decreased dietary sources of vitamin D	Dietary habits	
	Vitamin D replacement	
Newborns	<Correlation between vitamin D status in maternal and cord blood as measured by total circulating 25(OH)D concentration		

## 4. Discussion 

In this section, vitamin D status in mothers and their newborns and predictors for low maternal and neonatal (25(OH)D) concentrations in the Middle East will be discussed in detail.

### Vitamin D status in mothers and their newborns

A US-based study from California reported extensive vitamin D insufficiency and deficiency during pregnancy that particularly affected mothers

and their newborns (53) Our review revealed the prevalence of (25(OH)D) concentrations < 50 nmol/L in pregnant women and their newborns was over a wide range, but at minimum, more than a third-to-half of the women and their newborns had frank VDD, and more than 75% had either deficiency or insufficiency. Our findings of VDD in mothers and their newborns in the Middle East are consistent with the previous systematic review that had shown a high prevalence of hypovitaminosis D among pregnant women (9, 49, 102, 103). Results of this study are similar to other studies in the United States and Europe. In more recent reports, 18% of pregnant women in the UK (103), 42% in northern India (104), 61% in New Zealand (105), and 60-84% of pregnant non-Western women in the Netherlands (106) were reported to have serum (25(OH)D) concentrations < 25 nmol/L. Results of this study are consistent with the previous reports and indicate that there is a widespread problem of VDD that persists in mothers and their neonates in the Middle East. The rates of deficiency vary, reflecting the definitions of VDD and insufficiency used. In the studies cited, estimates were based on the different categorization of (25(OH)D) concentrations such as < 10, < 20, < 25, and < 35 nmol/L. There is no universally agreed upon definition of VDD and insufficiency (107).

According to the United States' Institute of Medicine, serum 25-hydroxyvitamin D (25(OH)D) concentrations < 50 nmol/L (20 ng/mL) are considered as low vitamin D status in adults and children (108). Based on clinical laboratory classifications, vitamin D insufficiency is defined as ≥ 50 to < 80 nmol/L (≥ 20 to < 32 ng/mL), and sufficiency as ≥ 80 nmol/L (≥ 32 ng/mL) (109-110). Zeghoud and colleagues proposed (25(OH)D) concentrations < 30 nmol/L (12 ng/mL) as the cutoff for diagnosing hypovitaminosis D in the newborn (111). It is generally accepted that concentrations of > 30 ng/mL are ideal, and concentrations < 20 ng/mL are considered deficient (112). The Institute of Medicine in the United States has indicated that serum (25(OH)D) concentrations > 50 nmol/L (or 20 ng/mL) are adequate for pregnant women (108); however, Holick (2009) and Dawson-Hughes and colleagues have defined optimal concentrations as > 75 nmol/L or 30 ng/mL (113). Hollis and Wagner in their randomized controlled trial of vitamin D supplementation in 350 pregnant women (110) found that the conversion of (25(OH)D) to (1,25(OH)${}_{2}$D) is optimized at a (25(OH)D) concentration of at least 100 nmol/L (40 ng/mL), the only time in the lifecycle that (25(OH)D) and (1,25(OH)${}_{2}$D) are so highly correlated. This is also found in the neonate at birth, but beyond the neonatal period this relationship no longer exists (114).

### Factors influencing vitamin D status in newborns including the correlation between 25(OH)D concentrations in maternal and cord blood 

Newborns receive their vitamin D via the placenta throughout pregnancy in the form of 25(OH)D (46). Eight studies in this review have shown a strong positive correlation between maternal and fetal circulating 25(OH)D concentrations (6, 47, 57, 67, 71, 73, 89, 93). The results of this review are consistent with other systematic reviews of studies reporting serum (25(OH)D) concentration in maternal and newborn populations in the Americas, Europe, Eastern Mediterranean region, South-East Asia, Western Pacific, and African countries (49). Most of the investigators have reported that there is a positive and strong correlation between maternal and cord blood (25(OH)D) concentrations (33, 110, 115-117), and this is supported by recent studies from many countries, including India (103), the United Kingdom (118), Greece (119), and the US (110, 117, 120). Not surprisingly, these studies further demonstrated a high prevalence of VDD in mother-infant pairs at birth.

### Factors influencing Vitamin D status in mothers 

#### Decreased vitamin D synthesis due to personal and environmental factors 

In this review, personal factors associated with decreased vitamin D synthesis included lifestyle (6, 47, 55, 76, 85), and cultural conditions (6, 76, 85, 86, 93, 101) due to greater coverage of the body with clothing that resulted in lower duration as well as less surface area exposed to the sun (121, 122), less participation in outdoor activities (123), and the use of sunscreen, which blocks UVB radiation, and thus vitamin D synthesis in the skin (124). Similar results were observed in studies in the Middle East and countries with Muslim communities (125-126). Women residing in the Middle East and Southeast Asia tend to spend very little time in the sunlight due to cultural and social reasons (127). Studies in Nigeria and Gambia have shown that VDD in newborns at birth is rare when mothers' exposure to sunlight is unrestricted (128). Decreased time spent outdoors may be related to increasing urbanization and increasing time spent indoors at work. Shade reduces the amount of solar radiation by 60% and windowpane glass blocks UVB radiation (123).

Countries of the Persian Gulf region—Bahrain, Iran, Iraq, Kuwait, Oman, Qatar, Saudi Arabia, and the United Arab Emirates—have become increasingly modernized, resulting in lifestyle transformation based on technology, sedentary activity, and lack of sunlight. These factors have led to a higher prevalence of VDD (129). Besides urbanization, there are multiple factors that limit sunlight exposure such as the hot environment/weather (130), social norms, and religious habits (129-131). Women avoid going outdoors due to more urbanized lifestyles and aesthetic reasons; for example, the culture tends to favor a fair skin that is not suntanned. Therefore, even in the privacy of their own homes, women from such cultural background tend to avoid sun exposure (130). This is in marked contrast to the more Western cultures where tanning and darkening of the skin pigment among white/Caucasian women are considered to enhance their beauty. Cultural norms thus play a role in the time spent outdoors and in the sun, and both directly affect vitamin D status.

As mentioned earlier, sun exposure is also greatly affected by clothing styles that cover most of the body, which leads to reduced vitamin D production in the skin (129). Thus, limited sun exposure in the Middle East appears to be mostly due to cultural practices, clothing styles and limited outdoor activity (132). Other factors such as cloud cover of the sun (133), atmospheric pollution (121, 133, 134), geographic latitude (46), and season (122) are environmental factors influencing the amount of UVB radiation reaching earth (127). In this study, factors associated with vitamin D status included air pollution (98, 73) and seasons (98, 101). Our data indicate that exposure to ambient urban air pollution during pregnancy can significantly contribute to VDD in newborns at the time of delivery. A cohort study in France reported an inverse correlation between air pollution and (25(OH)D) concentrations in cord blood (135). The association between air pollution and low vitamin D status has also been reported among children (133, 136) and women (137, 138). Historically, this was the basis of rickets in children living in industrialized Great Britain where air pollution prevented UVB from reaching beyond the coal and pollutant layer (139). Air pollution decreases vitamin D synthesis in the skin in two ways: Pollution reduces the amount of UVB radiation reaching the skin (121) and also the amount of time that people are outdoor (140).

In a study of newborns in Amman, Jordan, Khuri-Bulos *et al.*, reported that birth during winter months was associated with lower infant 25(OH)D concentrations (98). In a US cohort of 100 infants who were born at latitude 32°N, both the groups of African-American and Caucasian infants exhibited a seasonal variation in vitamin D status (46). An alternative explanation can be that when pregnancy was in winter, these infants accrued lower stores of vitamin D *in utero *(141). Other studies have shown that the prevalence of VDD during the winter months was higher than the summer months (119, 142-143). Vitamin D status is typically low in the winter months due to variation in cutaneous photosynthesis of pre-vitamin D at higher latitudes above 30° (144). In addition, in the winter the mothers use warmer clothing and the less body surface exposure reduces the cutaneous synthesis of vitamin D (46).

#### Decreased dietary sources of vitamin D due to dietary habits and vitamin D replacement

The results of this review study showed that another predictor for low 25(OH)D concentrations included decreased dietary sources of vitamin D due to dietary habits and lack of vitamin D replacement (85, 86, 93, 101). Dawodu and colleagues (2015), in the Global Exploration of Human Milk study, concluded that the prevalence of VDD in diverse populations appears to depend on sunlight exposure behaviors and vitamin D supplementation use (145). This is in support of the findings in this study. Vitamin D is a fat-soluble vitamin, which is found

naturally only in a few foods, such as fish-liver oils, fatty fish, egg yolks, liver, and mushrooms (146, 147). Among the dietary sources, the only ones that seem to affect 25(OH)D synthesis are fish (85) and mushrooms (147) if consumed in sufficient quantity. Promoting fish and mushroom consumption in pregnant women may be reasonable with respect to 25(OH)D concentration (85); however, with the former, there is concern about heavy metal exposure and the transmission of this to the developing fetus (148).

The currently recommended dose of vitamin D during pregnancy differs in different parts of the world. In the United States, the daily recommended dose by the Institute of Medicine is 400-600 IU/day (149), while the Endocrine Society suggests a daily dose of 2000 IU/day. In the UK, the recommendation is 400 IU/day (150), and in Turkey, it is 200 IU/day (86). There are other studies that suggest that the requirement for optimal vitamin D status during pregnancy is 4,000 IU/day (110). Based on this sytematic review that shows widespread deficiency throughout the Middle East in pregnant women, higher intake is likely necessary for vitamin D supplementation during pregnancy for women living in the Middle East (74) and those with an increased body mass index (151). Different studies suggest that the daily vitamin D intake during pregnancy should be 600 IU (152), 1,000 IU (112, 115, 153), 2,000 IU (107, 154), and 4,000 IU (109-110, 155-157), and that increased supplementation would help to eliminate vitamin D insufficiency without apparent toxicity in pregnant women and their infants. There is ample evidence that a daily dose of 4,000 IU/day vitamin D${}_{3}$ will safely achieve vitamin D sufficiency in more than 95% of women and the 100 nmol/L circulating concentration of 25(OH)D that is necessary for optimal conversion of 25(OH)D to 1,25(OH)${}_{2}$D (158-159).

## 5. Conclusions

The Middle East, in most of its geographic regions, has sufficient sunshine for the adequate photocutaneous synthesis of vitamin D; however, most of the Middle Eastern women cannot benefit from this source because of cultural practices, such as clothing and veiling among Muslim women, and spending most time indoors. The results of this study raise the concern that newborns in the Middle East are entering the world with a vitamin D deficiency that begins *in utero*, with documented consequences on later health such as low birth weight, small-for-gestational age (7, 9), abnormal skeletal development, type 1 diabetes (35), abnormal immune development (37), wheezing, asthma (40), abnormal neurocognitive development (41), and the concern for epigenetic factors that can adversely affect health later in life (160).

The high prevalence of hypovitaminosis D in the Middle East underscores the necessity of implementing national preventive strategies. These strategies for the prevention of VDD in women and their newborns at birth begin with vitamin D sufficiency in women during pregnancy. The preventive strategies need to encompass public awareness and also guidelines for health care personnel concerning screening and supporting vitamin sufficiency (161). In most countries, there is no routine screening for VDD nor for insufficiency during pregnancy. A 2013 study and 2009 review have recommended that women with one or more risk factors for low serum 25(OH)D should be screened at the beginning of gestation and in mid-pregnancy and early in the antenatal period, especially for those with risk factors (47, 116). Such screening programs already have been implemented in centers in the US with a positive effect (162).

Based on this current view, more than 75% of Middle Eastern women are either frankly vitamin D deficient or insufficient, which directly reflects their reduced exposure to sunlight and decreased dietary sources of vitamin D, thus making them significantly more likely to have lower 25(OH)D concentrations. These findings lend support to a screening of pregnant women and their newborns for VDD in all Middle Eastern women. If a mother has VDD or insufficiency and sunlight exposure is limited, an alternative for the pregnant woman is vitamin D supplementation during pregnancy to ensure vitamin D sufficiency for both herself and for her newborn (33, 163). Daily doses of 4,000 units/day are recommended for achieving vitamin D sufficiency throughout pregnancy (110, 164). Because vitamin D status is inversely associated with obesity parameters, women with higher BMIs will need higher doses of vitamin D supplementation (165-167). Dietary intake alone is not sufficient to maintain normal vitamin D status because a considerable amount of vitamin D is acquired by cutaneous synthesis when exposed to sun (121, 137). Therefore, public health awareness about the predictors of low maternal and neonatal 25(OH)D concentrations in the Middle East and also the need to encourage vitamin D sufficiency by modest sunshine exposure and adequate maternal vitamin D intake during pregnancy are needed.

##  Strengths and Weaknesses of the Study

A strength of our study was the use of a comprehensive search strategy with broad search terms. A potential weakness is that we restricted the review to studies published in the English language in two international electronic databases (Scopus, PubMed), so relevant studies may have been missed.

##  Conflict of Interest

The authors declare that they have no competing interests.
